# Digital Platform for Wafer-Level MEMS Testing and Characterization Using Electrical Response

**DOI:** 10.3390/s16091553

**Published:** 2016-09-21

**Authors:** Nuno Brito, Carlos Ferreira, Filipe Alves, Jorge Cabral, João Gaspar, João Monteiro, Luís Rocha

**Affiliations:** 1 Algoritmi Center, University of Minho, Guimarães 4800-058, Portugal; danielferreira65350@gmail.com (C.F.); jcabral@dei.uminho.pt (J.C.); joao.monteiro@dei.uminho.pt (J.M.); 2 CMEMS-UM, University of Minho, Guimarães 4800-058, Portugal; id4465@alunos.uminho.pt (F.A.); lrocha@dei.uminho.pt (L.R.); 3 INL, International Iberian Nanotechnology Laboratory, Braga 4715-330, Portugal; joao.gaspar@inl.int

**Keywords:** microelectromechanical devices, microprocessors, field programmable gate arrays

## Abstract

The uniqueness of microelectromechanical system (MEMS) devices, with their multiphysics characteristics, presents some limitations to the borrowed test methods from traditional integrated circuits (IC) manufacturing. Although some improvements have been performed, this specific area still lags behind when compared to the design and manufacturing competencies developed over the last decades by the IC industry. A complete digital solution for fast testing and characterization of inertial sensors with built-in actuation mechanisms is presented in this paper, with a fast, full-wafer test as a leading ambition. The full electrical approach and flexibility of modern hardware design technologies allow a fast adaptation for other physical domains with minimum effort. The digital system encloses a processor and the tailored signal acquisition, processing, control, and actuation hardware control modules, capable of the structure position and response analysis when subjected to controlled actuation signals in real time. The hardware performance, together with the simplicity of the sequential programming on a processor, results in a flexible and powerful tool to evaluate the newest and fastest control algorithms. The system enables measurement of resonant frequency (Fr), quality factor (Q), and pull-in voltage (Vpi) within 1.5 s with repeatability better than 5 ppt (parts per thousand). A full-wafer with 420 devices under test (DUTs) has been evaluated detecting the faulty devices and providing important design specification feedback to the designers.

## 1. Introduction

Test and characterization of microelectromechanical systems (MEMS) is a mandatory step towards the improvement of both quality control and design iteration processes. Industry studies state that the expenses associated with the test and characterization of manufactured MEMS devices takes a significant share of the total production cost [1,2,3].

The optical and mechanical tests for MEMS characterization, although precise, are not well suited for a fast, full-wafer diagnosis. The use of electrical stimulus, using a fully electrical test, can provide a fast, cost-wise, and easy-to-adjust system as a step forward in MEMS testing, characterization, automation, and cost reduction.

The full testing of sensors such as accelerometers and gyroscopes poses a challenge since it requires the devices’ exposure to physical stimuli, in order to evaluate the mechanical characteristics. The current approaches for early detection of manufacturing issues are traditionally based on testing and sampling random wafer structures. Since the process is slow, a full-wafer test is expensive and time consuming [4]. Modern industry standards use highly sophisticated test platforms with high-throughput to test all the manufactured devices, but these systems are designed for a specific set of requirements and traditionally kept undisclosed from the scientific community.

The electrical test is a technique initially borrowed from the traditional IC industry, in which a microprobe is used to inject electrical signals into the structure and another probe is used to measure the resulting electrical signal from which some mechanical properties can be derived. The low-latency possibilities offered by this technique [5,6], the possibility to test a large range of sensitivities of devices [6], and the lack of need for using costly mechanical stimulation are good arguments to develop this approach. This traditional IC technology still needs to be further enhanced to adjust to MEMS multiphysics behavior, which is still in an early development phase, in comparison to the well-studied manufacturing and design efforts [1].

As an alternative approach, the optical technologies, conducted by companies such as Polytec [7], consists of using light-interferometry to measure the structure vibration velocity and displacement information, which is then used to infer the structure mechanical properties. Other approaches, such as [8], use optical microscopy associated with image-processing algorithms to measure the device depths, sizes, and cavities. An alternative to these tests is the electromechanical testing provided by tools such as the FT-MPS02 MEMS probe station by FEMTOTOOLS [9], where a nanoposition platform and a sensing microprobe can be used to physically apply forces to the structure and evaluate the actual system response. One way to reduce testing time using existing methods is by increasing the throughput by testing multiple devices in parallel. However, these horizontal scalability techniques require extra equipment and present their own challenges and limitations [10,11].

In this article the development of a flexible and modular MEMS testing and characterization prober based on a processor-centric hardware-assisted embedded solution, using the built-in electrostatic actuation mechanisms, is presented. The dedicated hardware digital system is responsible for excitation, measurements, filtering, and real-time control loops necessary for analysis of the MEMS devices, and greatly increases speed and flexibility of the test. On one hand, the system enables measurement of resonant frequency (Fr), quality factor (Q), and pull-in voltages (Vpi) in 1.5 s (3–7 times faster than the results presented in [5]) while on the other hand, the use of a softcore processor enables a high degree of flexibility, making it possible to quickly adapt or change the testing algorithms for different devices, as opposed to using fully hardware implementations, as is the case in [5].

The system was used to test individual MEMS inertial devices, and was also tested with a semiautomated probe station for testing and characterization of MEMS at the wafer-level. The detection and evaluation of process-dependent variables, like over-etching in the production cycle, are achieved and presented. The preliminary results of the system using an analog readout have been presented in [12]. The platform has since been improved by switching to a complete digital processing solution that is based on a lock-in amplifier, and testing has been extended to full-wafer characterization.

The article is divided in 4 sections. After an Introduction, the design and implementation of the digital platform is presented in the second section. Next, in the third section, MEMS characterization results are presented for both individual MEMS and wafer-level testing. Finally, some conclusions are presented regarding the suitability of the presented approach for MEMS testing, characterization, and process monitoring.

## 2. Flexible and Modular Digital Platform

### 2.1. MEMS Devices Specification

**Figure 1:**
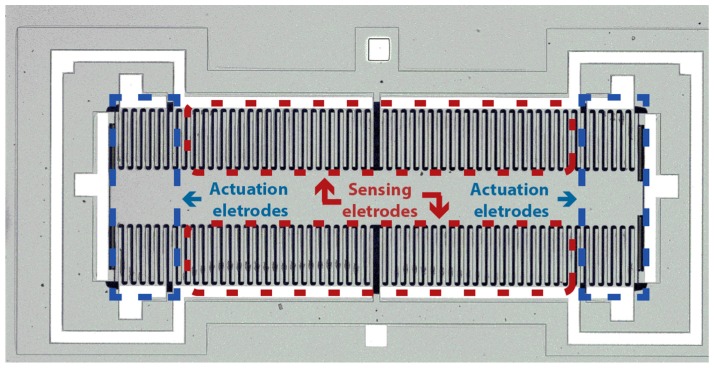
Capacitive microelectromechanical system (MEMS) sensor with sensing and actuation electrodes highlighted areas.

The digital platform for MEMS evaluation presented here is a fully electrical characterization solution for electrostatically actuated MEMS accelerometers with capacitive readout (it can nevertheless be easily adapted to other transduction mechanism, by changing the readout block). The core principle is the ability to use electrostatic actuation on the structure actuation electrodes and then evaluate the structure displacement by measuring the capacity variations through a charge amplifier. Figure 1 presents a picture of one of the used MEMS inertial sensors, clearly showing the electrodes used to electrostatically actuate on the structure (enabling bidirectional actuation) with the sensing electrodes in the middle region.

It has already been demonstrated that inertial MEMS sensors can be fully described by their resonance frequencies (Fr), quality factors (Q), and pull-in voltages (Vpi) [13,14]. In addition, these characteristics can also be used to access process-related variations like over-etching and material properties (Young’s modulus and density, for instance). These are key device characteristics and their fast and accurate measurement can be very important for both MEMS designers (to evaluate device performance during the design phase) and for process monitoring and/or process development. Therefore, the MEMS testing and characterization prober presented here aims the acquisition of these parameters using a fast and reliable architecture.

Although the system was developed to test one-axis electrostatically actuated capacitive accelerometers, the digital system can be adapted to other types of devices that can be electrically stimulated by programming the specific digital circuits.

### 2.2. Overview

**Figure 2:**
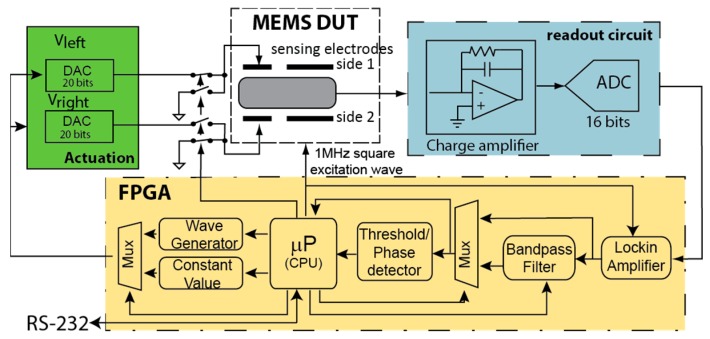
Block diagram of the MEMS test and characterization system, including hardware- and software-embedded blocks. (DUT: device under test; FPGA: Field programmable gate array).

The flexible digital platform, schematically presented in Figure 2, uses an ADC and a charge amplifier to convert the device capacitive changes to voltage (the only analog circuit), a central processing unit, and a 2-channel digital-to-analog converter (DAC) board to inject signals. The digital processing system is developed and implemented in an FPGA (field programmable gate array) using the Leon 3 softcore microprocessor [15], along with custom-designed peripherals enabling true parallel and deterministic behavior, which are necessary to meet the application requirements for fast and accurate testing. The interrupt-driven nature of the digital peripherals allows for true parallel real-time processing capabilities and scalability because it lays on the hardware for the heavy processing that a software system cannot attend.

Due to the flexibility of the system, based on a softcore microprocessor, other measurements (besides the resonance frequency, quality factor, and pull-in voltages) can easily be implemented and added to the system by programming the microprocessor. The digital blocks, implemented in hardware, are presented next. While the blocks that are responsible for the MEMS characteristics acquisition (pull-in measurement, resonant frequency measurement, and quality factor measurement) are novel and advance the state-of-the-art in the field. The remaining blocks, although important (like the lock-in and filters) are just implementations of existing technologies/methodologies.

### 2.3. Lock-in Amplifier

The capacitive transduction in MEMS allows a higher sensitivity than a piezoresistive equivalent [16] with the cost of more complex electronic circuits. The capacity measurement is then an important block in order to obtain the displacement signal from the MEMS device. The MEMS platform was designed with a single analog block and a charge amplifier, followed by a digital lock-in amplifier allowing a digital tuning of the amplifier gain and improved noise performance (through the implementation of high-order digital filters).

Digital lock-in amplifiers [17] are circuits used to detect and measure very small alternating current (AC) signals buried in noise. Since it consists of digital multiplication and filtering, it eliminates the need for a noisier analog amplifier, as well as allows digital tuning, as opposed to the fixed analog components of traditional amplifiers. The signal frequency is isolated and all the other frequencies are frequency-shifted and filtered out, making it suited for noisy environments.

**Figure 3:**
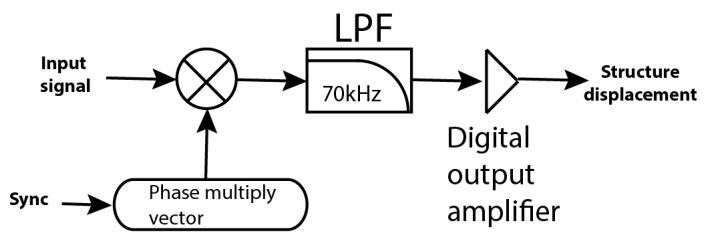
Digital lock-in amplifier logic blocks. (LPF: Low-pass filter).

In this work, two 1 MHz excitation signals (in phase opposition) are applied to the MEMS differential sensing capacitors. After conversion of capacitive changes to voltage (amplitude modulation) in the charge amplifier, the signal is digitally converted (at 5 MHz sampling frequency) and enters the digital lock-in amplifier block. The basic blocks of the lock-in amplifier are shown in Figure 3.

The generated excitation signals are used to synchronize the digital multiplication. After multiplication, a digital low-pass-filter with a corner frequency at 70 kHz removes the carrier, and digital gain can be used at the digital output amplifier. At the output of the lock-in there is a signal proportional to the MEMS displacement.

### 2.4. Pass-Band Digital Filter

**Figure 4:**
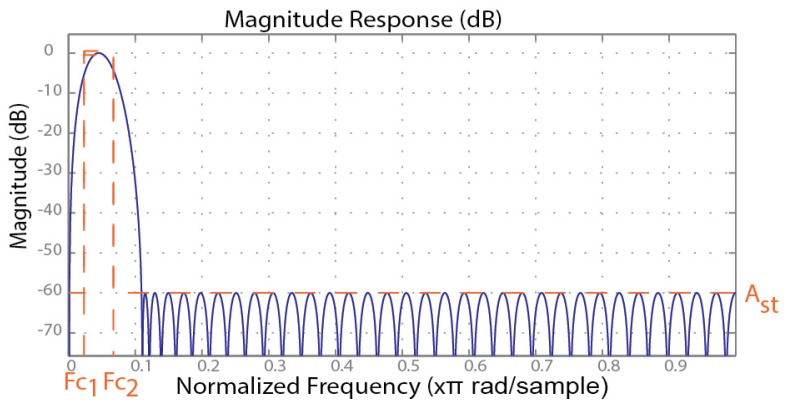
Band-pass finite impulse response (FIR) filter adjusted by changing the sampling frequency.

Taking advantage of the FPGA parallel computing capabilities, a 70 taps finite impulse response (FIR) filter is built to allow filtering the lock-in output signal according to the detection routine necessities. The processor can then perform a setup for the FIR coefficients. For instance, a band-pass (Fc1 = 0.023; Fc2 = 0.067; Ap = 1 db; Ast = −60 db) was designed and is used during the resonant frequency search procedure. Figure 4 shows the bode plot for the filter response to the normalized frequency, where the direct current (DC) voltage is rejected and the band-pass gain is close to 0 dB. The filter has a built-in sample prescaller that enables an easy adjustment of the filter by changing the sampling frequency, thereby enabling the change of the band-pass region along the frequency domain, which avoids changing the filter characteristics. This allows for a faster analysis, a predictable filter behavior, as well as a constant filter delay.

The filter phase delay was measured by applying a low frequency sine wave (structure delay ≈ 0) on the structure and measuring the delay between the DAC peak and the measured peak.

### 2.5. Peak/Threshold Detector

A programmable peak or threshold value detector with interrupt capabilities is available to the processing system. This peripheral is used to detect the movement peaks, which is then used to measure the structure delay. The threshold detector is used to detect the pull-in voltage and promptly notify the processor to take the actions (in this case to remove actuation) in order to avoid collisions.

### 2.6. Communications Interface

A standard RS-232 port is available and a simple protocol was developed to allow the automation by an external computer. This feature can be used to automate the whole process using an automatic probe station to move the wafer, apply the probes on the structure, and then call the test procedure as an independent black box.

### 2.7. Pull-in Measurement

**Figure 5:**
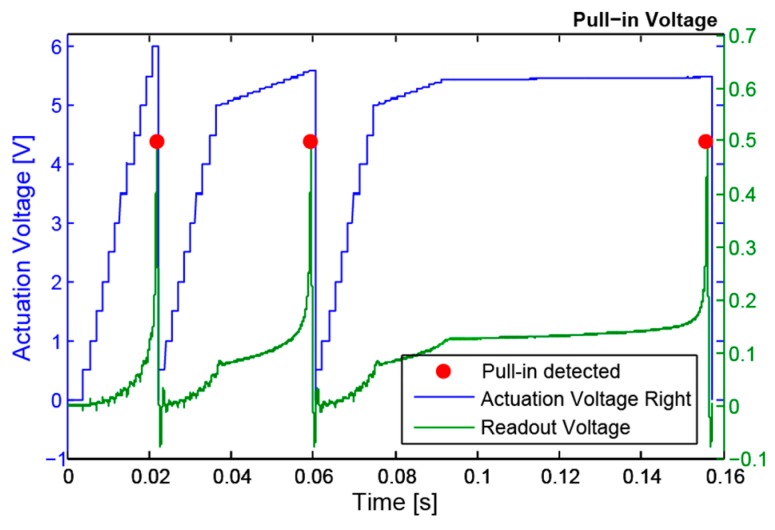
Pull-in ramp with highlighted detection points. The immediate actuation voltage removal is evident to avoid hitting the counter electrodes.

Pull-in voltages are an important characteristics of parallel-plate electrostatic actuators and can be used for device characterization [14]. The pull-in voltage is usually measured by looking at the device displacement while increasing the actuation voltage from zero until an abrupt change is detected. The voltage at which this abrupt change is detected corresponds to the pull-in voltage. It is therefore obvious that the resolution of the measurement depends on the resolution used for the actuation voltage increase. In this work, and in order to have an acceptable resolution (1 mV resolution as used in [15]), pull-in voltages are measured by applying three consecutive ramps with increasing resolution (500 mV, 40 mV, and 1 mV) as demonstrated in Figure 5. Such an approach allows the algorithm to jump with a larger resolution to a starting point closer to the pull-in voltage. This feature strongly speeds up the pull-in voltage measurement (if a 1 mV ramp would be used from 0 until pull-in is detected, the required time to measure pull-in would be considerably more). This procedure is repeated at least twice for each side to increase robustness, i.e., only after two consecutive similar pull-in voltages are measured is the measured pull-in voltage considered valid.

Another key feature of this block is the displacement detector that prevents the device from hitting the counter-electrodes. During pull-in measurements, the peak/threshold detector (Section 2.5) is programmed to trigger an interrupt on the processor when the device displacement is larger than 1/3 of the gap (when pull-in occurs). When the interrupt is triggered, the microcontroller switches off the actuation voltage and the device returns to its rest position, preventing it from hitting the stoppers. This interrupt also informs the microcontroller that pull-in was reached and the voltage applied is stored as the corresponding pull-in voltage.

Figure 5 shows a real example of the described ramp generation method with the pull-in detection points highlighted and where the delay between the detection and the voltage removal, which is 1 ms long, can be seen.

### 2.8. Resonant Frequency Measurement

**Figure 6:**
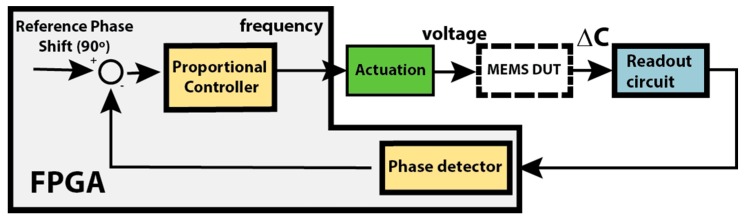
Resonant frequency proportional controller blocks.

A characteristic of second-order systems is that at resonance, the system output is 90° phased-shifted with the input. In this block, the resonant frequency is measured by injecting a sine wave at a known frequency on one of the structure actuation electrodes while the other side’s actuation electrode is actuated in phase opposition (both sine waves have a DC-positive offset). This configuration [18] enables the movement of the structure with a frequency equal to the one applied, eliminating the squared terms from the electrostatic force. Since the device frequency displacement is known, the digital band-pass filter is then digitally adjusted to the actuation frequency, strongly eliminating noise, and the phase difference between actuation and displacement is measured. This procedure can be performed for several frequencies to retrieve the device bode plot, but since the goal is to have a fast characterization setup, a proportional controller (Figure 6) was implemented that automatically adjusts the applied frequency (and filter parameters) until a 90° phase shift is achieved (at the resonance frequency). The use of the controller is critical for a fast determination of the resonance frequency.

### 2.9. Quality Factor Measurement

The quality factor is an indicator of the system’s gain at resonance. Therefore, after resonance frequency measurement, the quality factor is measured by using the ratio between the displacement amplitude at the resonance frequency and displacement amplitude one decade before (amplitude measured during two periods). This procedure is repeated five times for increasing the system robustness and the quality factor is taken as the average of the five measurements.

## 3. Digital Platform Characterization and Testing Results

**Figure 7:**
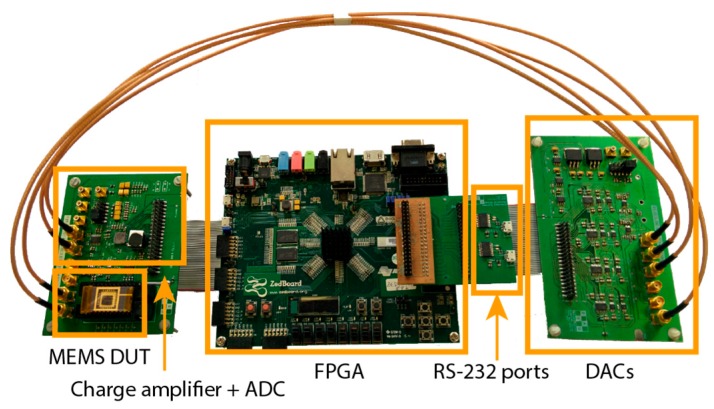
Image of the prototyped digital platform.

Initially, the main blocks of the digital platform were characterized, followed by the test and characterization of MEMS devices both individually and at the wafer level. An image of the digital platform, where the main blocks are identified, is presented in Figure 7. On the left, the MEMS device and readout circuitry custom-developed are shown. The center block is the off-the-shelf Zedboard FPGA development kit. The right module is a custom-developed 4-channel DAC board (only two channels are used in the current work).

### 3.1. Capacitive Readout Characterization

**Figure 8:**
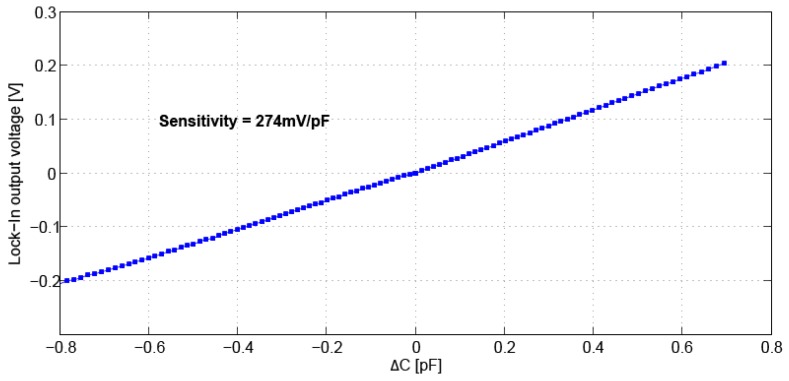
Output response of the capacitive readout block, including digital lock-in.

A key performance block in this application is the capacitive readout system. The full readout system, including the digital lock-in amplifier, was characterized using a known MEMS device with 1-DOF (degree of freedom). Since pull-in occurs in 1-DOF structures at 1/3 of the total gap [14], voltages were applied to the structure and the correspondent voltage changes measured by the platform (that can be translated to displacement and to capacitance changes knowing the device design) were recorded. The device sensitivity (S = 274 mV/pF) and linearity (1.3% FS with FS = ±700 fF) were than evaluated, and the result is presented in Figure 8.

**Figure 9:**
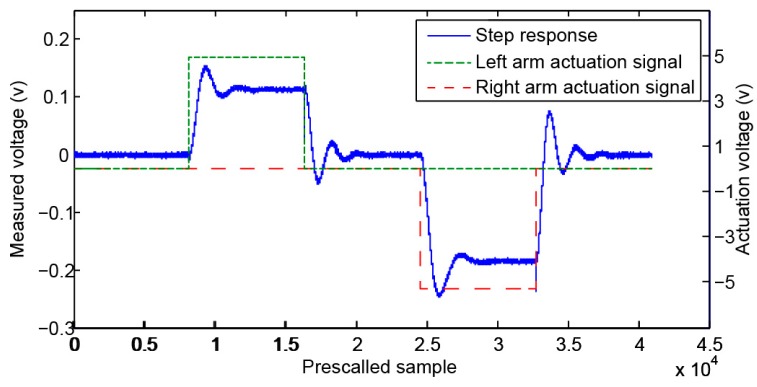
Step responses of the structure.

One advantage of the digital platform developed is the simplicity and flexibility to develop custom routines. Figure 9 shows the measured response to a right-arm and left-arm steps, where the oscillations of the DUT (device under test) are visible, showing that in this case we are in the presence of an underdamped device (Q > 0.5).

Further measurements were performed to characterize the total noise of the capacitive readout block (including the lock-in) and the total noise has been found to be 6.2 µV/√Hz, which translates to a capacitive detection of 22.6 aF/√Hz.

### 3.2. Single Devices Test and Characterization

**Figure 10:**
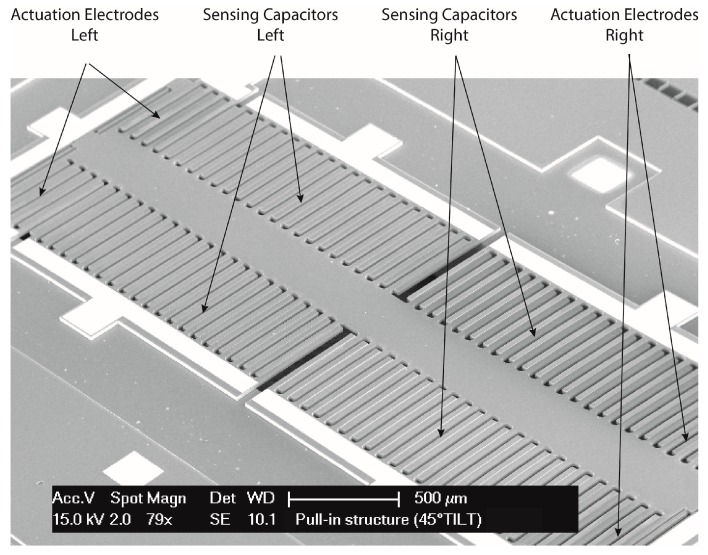
SEM image of a MEMS DUT.

After characterization of the capacitive readout block, several MEMS accelerometers were tested and characterized. An SEM image of a typical device is shown in Figure 10. All the characterized devices enable electrostatic actuation (along one plane direction) and have a differential capacitive readout for displacement transduction.

**Figure 11:**
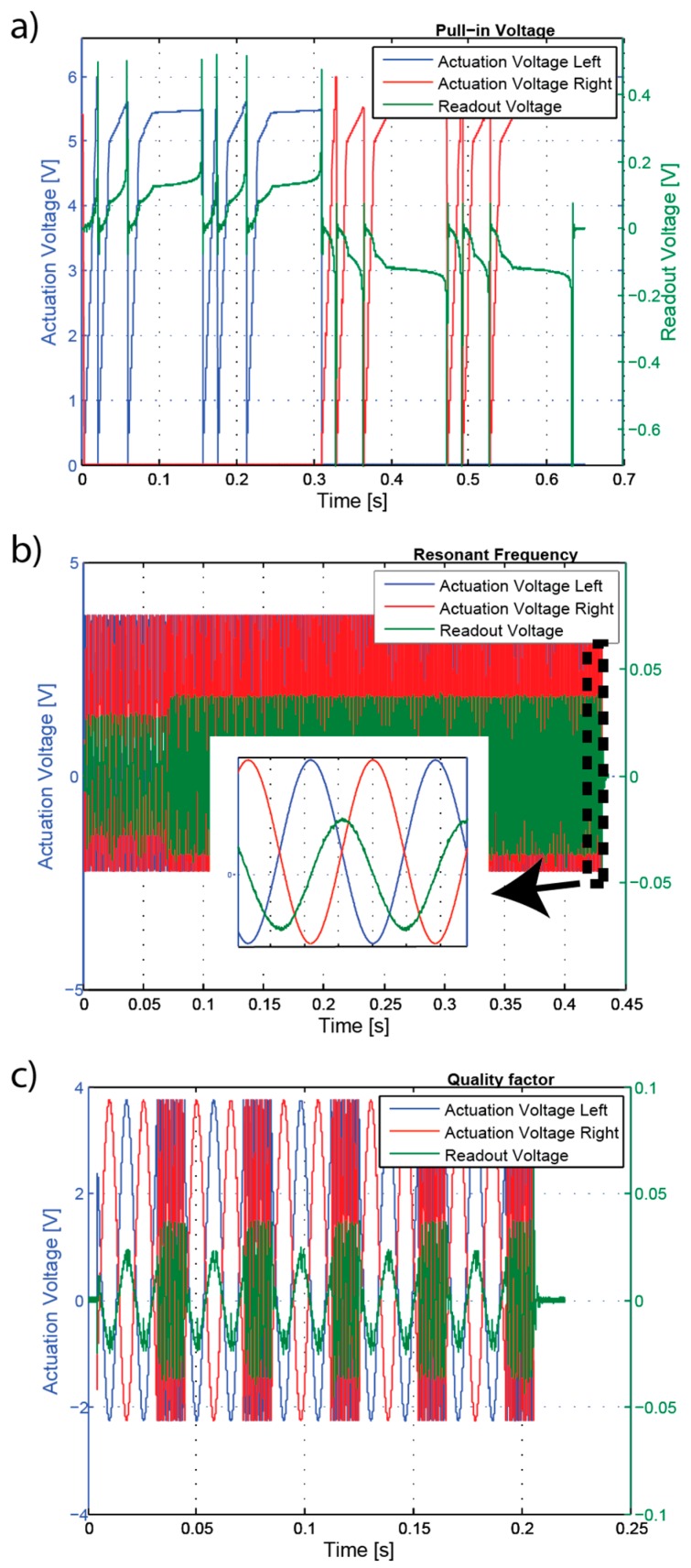
Full measurement cycle overview (actuation and voltage readout) (**a**) Pull-in voltages measurement cycle; (**b**) resonant frequency measurement cycle with inset showing 90° phase shift at resonance frequency; and (**c**) quality factor measurement cycle.

Figure 11 shows a complete measurement cycle where the actuation voltages and measured response are presented. Figure 11a shows the pull-in voltage measurement cycle. As described before, for robustness increase the pull-in voltage measurement is repeated until two consecutive pull-in voltage measurements are equal, and therefore the 12 ramps (3 per cycle, 2 cycles for each side). Figure 11b presents the resonance frequency measurement. The inset shows the stop criteria when the signal output is 90° phase-shifted with respect to the input actuation voltages. Figure 11c shows the measurement of the quality factor. The amplitude of the signal output is measured at the resonance frequency and one decade before, and quality factor is calculated as the ratio between these two amplitudes. The procedure is repeated five times. The required time for each measurement can be taken from the x-axis.

**Figure 12:**
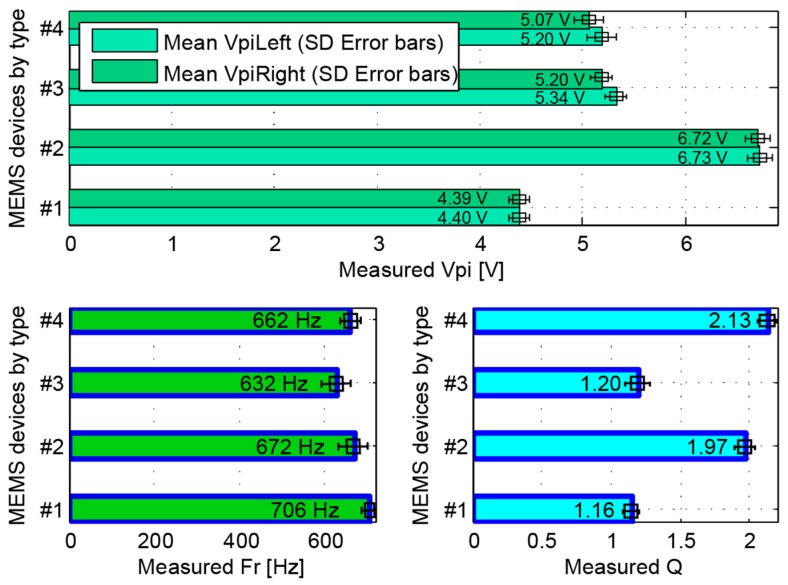
Characterization values for four different types of devices, capturing the design differences between devices.

Figure 12 shows characterization results for different type of devices where their design differences are clearly captured. In this test, a total of 11 devices (type #1—4, type #2—2, type #3—2 and type #4—3) were used. Type #1 and Type #2 have similar spring and mass with Type #2 having fewer capacitor arms (higher pull-in and quality factor) and Type #3 and Type #4 have exactly the same geometry, but Type #4 uses a damping reducing mechanism [19] and therefore has higher quality factor.

Repeatability of the measurements was also performed for one device and the results of 2600 measurements for each characteristic are shown in Table 1. The results show that full mechanical characterization is obtained in less than 1.5 s without any algorithm optimization.

### 3.3. Wafer-Level Testing Setup

Testing and characterization at the wafer-level is very important since relevant information regarding the process variability within the same batch, and critical information about the process (that can later be used to improve the design) can be derived. Therefore, the availability of a fast and reliable characterization system for MEMS at the wafer-level can be of utmost importance during the development phase (device design and process development), but also for production and calibration.

**Figure 13:**
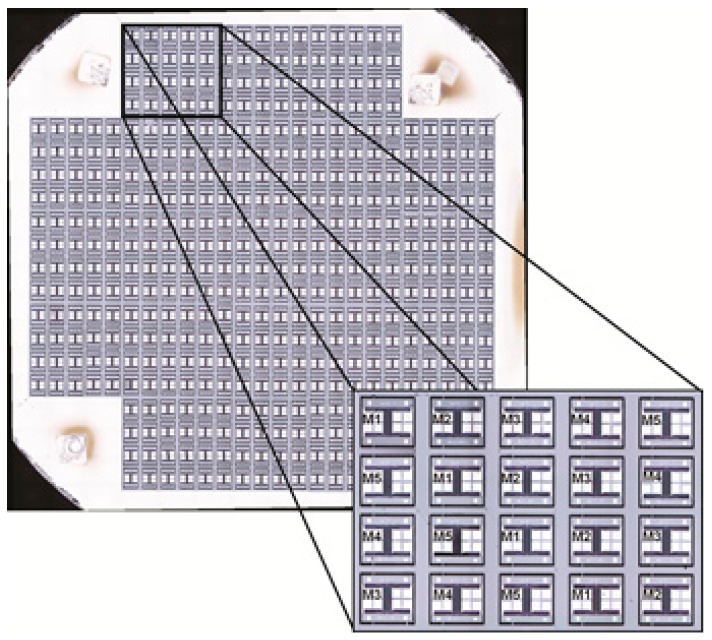
A 200 mm processed silicon-on-insulator (SOI) wafer with 21 reticules (420 DUT). Each reticule contains 20 MEMS devices (five different configurations, M1–M5).

**Figure 14:**
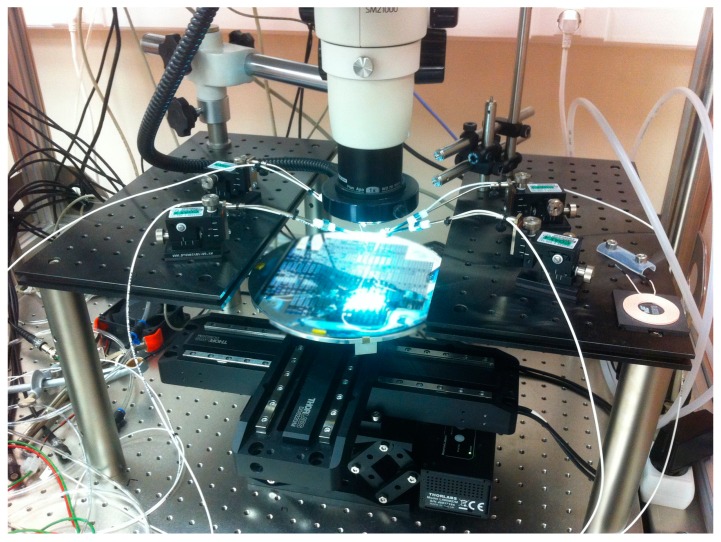
Semiautomated probe during wafer-level testing.

The digital platform developed was used to perform tests at the wafer-level. A 200 mm processed silicon-on-insulator (SOI) (25 µm active layer) wafer containing 420 MEMS devices (with five very similar layouts, M1–M5 distributed in 21 reticules, as shown in Figure 13) was tested using a semiautomated probe station (Figure 14). Electrical probes were used to connect the MEMS actuation electrodes and the sensing capacitors to the digital platform. The existing control software for the probe station allows loading the testing points along the wafer, but since the software is closed (does not enable external triggering), it was not possible to link the probe station control software with the digital platform RS-232 serial interface. Therefore, every time a new position was available for test, a manual input was required to start the MEMS testing, which significantly increased the total testing time.

For the wafer-level testing, a new simple routine to check if the device is good or faulty was added to the digital testing platform, which is performed prior to the characterization of the resonance frequency, quality factor, and pull-in voltage. This routine applies a known excitation voltage while checking the device displacement. In case the device moves above a predefined threshold (meaning the device is moving), it performs the remaining characterization; otherwise, it passes to the next device and signals that the device as faulty.

### 3.4. Wafer-Level Testing Results

For all 420 DUTs that were tested, data was recorded regarding the device status (good or faulty), and the measured mechanical characteristics (resonance frequency, quality factor, and pull-in voltage) for the good devices. The information was then post-processed in order to analyze process variability and process characteristics like over-etching. For the calculation of the process over-etch, the methodology presented in [15,20] was used. The method relies on accurate device models that include process variables (like over-etching). The measured data is then introduced in the models, enabling retrieval of the process variables.

**Figure 15:**
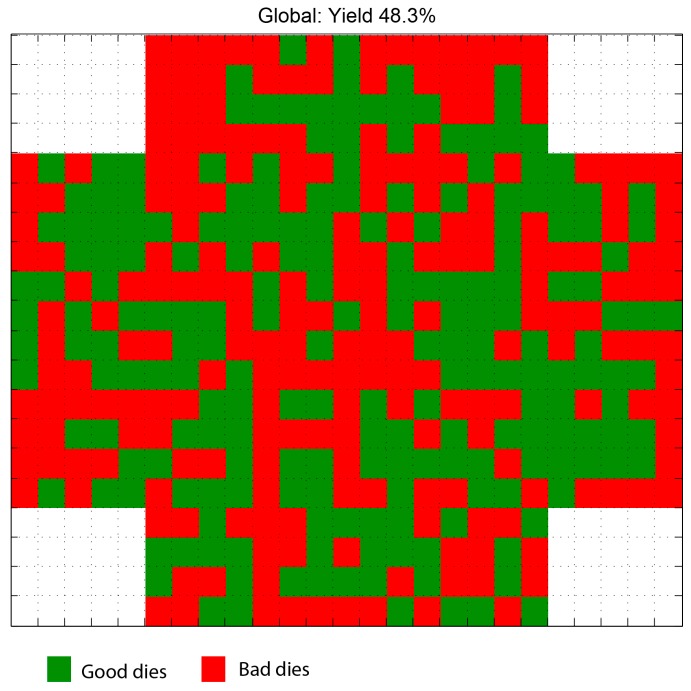
Results from the process yield.

The first analysis performed with the measured data was a yield analysis and location of the good DUTs within the wafer (see Figure 15). For the wafer analyzed, a global yield of 48.3% was obtained.

**Figure 16:**
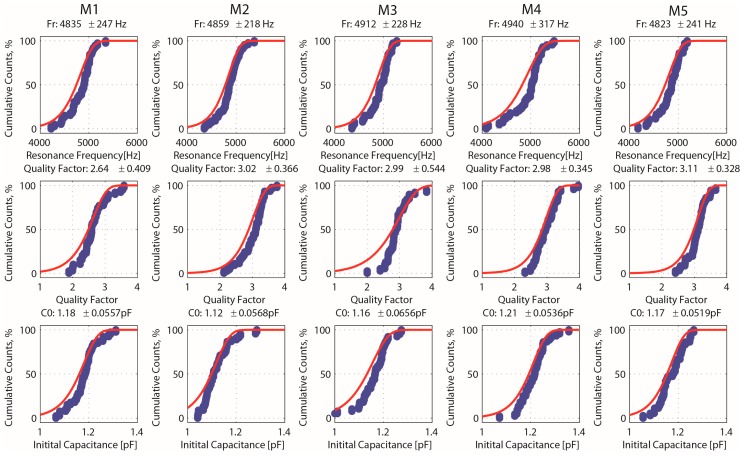
Cumulative distribution curves for resonance frequency, quality factor, and initial capacitance for the five different layouts (M1–M5).

Next, the process variability for the measured characteristics was plotted for the five different layouts (M1–M5). The cumulative distribution curves for the several parameters, average values, and standard deviation are presented in Figure 16. Overall, the measured parameters follow a Gaussian distribution within the wafer, and the small differences among the five different layouts are captured by the values measured. For comparison, the expected characteristics for the five different layouts are presented in Table 2, assuming a 1 µm over-etch, and are in good agreement (in average) with the measured values. Process uniformities around 30%, 60%, and 20% are found for the several layouts regarding the resonance frequencies, quality factor, and initial capacitance values, respectively.

**Figure 17:**
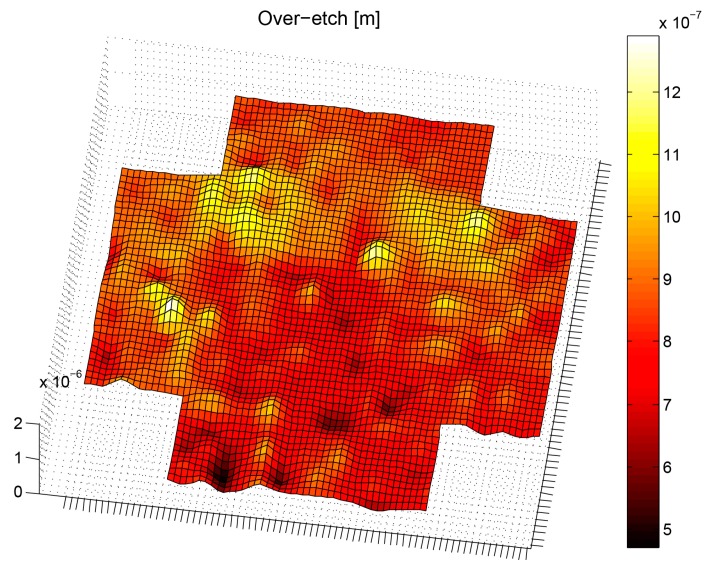
Over-etch wafer map.

Finally, the process over-etching was estimated for each device, and based on the device location within the wafer, an over-etching map was created as shown in Figure 17. An average over-etch of 840 nm was estimated from the measurements.

## 4. Conclusions

The digital platform for MEMS testing and characterization presented here is a cost-wise tool that can effectively be used for device and process characterization. Improved noise immunity was achieved with the digital lock-in amplifier which allows for a more accurate and fast characterization.

Wafer-level testing was performed and accomplished with good results. The three measured characteristics enable the detection of several production and design issues, and it is possible to perform a full-wafer test within a tolerable amount of time. Since the platform is fundamentally a digital implementation, several parallel channels can be connected, enabling the simultaneous measurement of devices (strongly decreasing the testing time). For instance, for a characterization time of 1.5 s (time experimentally validated) and a similar overhead time for automatically changing the electrical probes to a new device, the testing of a full-wafer with 420 DUTs would require 21 min. If 10 parallel characterization digital channels would be used, the total time would be reduced to 2.1 min.

Experimental data presented validates the digital approach followed in this work and shows that the digital platform is a powerful tool for MEMS testing and characterization. When combined with device modeling, it can be used for layout validation, during the design phase, process variability characterization, and process parameters estimation, such as the case of over-etching. Moreover, since the key mechanical characteristics are obtained, such characterization techniques can be used to derive calibration parameters for the MEMS devices. In the future, the algorithms used to measure the device characteristics can be optimized, in order to further reduce the testing time.

## Figures and Tables

**Table 1:** Measurement repeatability for 2600 samples (mean ± std).

Measurement	Value	Time (s)
Pull-in left [V]	5.477 ± 0.002	0.64 ± 0.04
Pull-in right [V]	5.415 ± 0.002	
Resonant Frequency [Hz]	620 ± 3.5	0.65 ± 0.36
Quality factor	1.76 ± 0.008	0.21 ± 0.01
**Total Time**		**1.5 ± 0.41**

**Table 2:** Expected parameters of devices M1–M5 based on layout, assuming 1 µm over-etch.

Device	M1	M2	M3	M4	M5
Expected resonance frequency (Fr) [Hz]	4794	4687	4793	4853	4751
Expected quality factor (Q)	3.17	3.37	3.39	3.36	3.57
Expected initial capacitance (C_0_) [pF]	1.10	1.06	1.09	1.14	1.11
